# New occurrences of mosquitoes (Diptera: Culicidae) in the Atlantic Forest biome of the Brazilian Northeast

**DOI:** 10.1590/0037-8682-0513-2024

**Published:** 2024-02-05

**Authors:** Íttalo Luã Silva Medeiros, Cinara Wanderléa Felix Bezerra, Mario Antonio Navarro-Silva

**Affiliations:** 1 Universidade Federal do Paraná, Programa de Pós-Graduação em Zoologia, Laboratório de Morfologia e Fisiologia de Culicidae e Chironomidae, Curitiba, PR, Brasil.; 2 Universidade Federal do Paraná, Programa de Pós-Graduação em Entomologia, Departamento de Zoologia, Curitiba, PR, Brasil.

**Keywords:** Immature mosquitoes, Larval habitats, Larval bionomy, Vector competence, Taxonomy

## Abstract

**Background:**

Information regarding the distribution of Culicidae species in the northeastern region of Brazil is scarce.

**Methods:**

Immatures were collected from approximately four fragments of the Atlantic Forest.

**Results:**

This study presents new occurrences of 18 Culicidae species in Pernambuco state: *Anopheles kompi*, *Georgecraigius fluviatilis*, *Culex bidens*, *Culex chidesteri*, *Culex bastagarius*, *Culex imitator*, *Mansonia humeralis*, *Wyeomyia incaudata*, *Uranotaenia apicalis*, *Culex mollis*, *Culex usquatus*, *Culex dunni*, *Culex serratimarge*, *Culex ybarmis*, *Culex microphyllus*, *Sabethes purpureus*, *Wyeomyia pilicauda,* and *Wyeomyia airosai*. The last nine species were also new records for the northeast region.

**Conclusions:**

With the inclusion of these newly recorded species, the total number of mosquitoes documented in Pernambuco state now rises to 94.

Expanding our knowledge on Culicidae diversity, particularly considering the blood-feeding behavior of females on vertebrate hosts, remains crucial. This behavior poses a potential risk of transmitting disease-causing agents to both humans and animals. Despite the significance of Culicidae in public health, some regions of Brazil, such as the Northeast, lack comprehensive information about their fauna^1^ ^),(^ ^2^ ^),(^ ^3^. Several factors hinder scientific studies on the biodiversity of insect vectors in the country, including geographical isolation, harsh climatic conditions, and limited research funding^4^. Consequently, accessing local biodiversity and identifying potential disease transmission pathways are fundamental for determining at-risk areas and implementing monitoring or control measures.

Historically, few studies have addressed the ecology of Culicidae in northeastern Brazil, leaving significant gaps in knowledge about the central areas of the Caatinga and Atlantic Forest biomes^5^ ^),(^ ^6^ ^),(^ ^7^. Moreover, investigations into the presence of immature studies within larval habitats have been limited^7^ ^),(^ ^8^. This study aimed to describe new occurrences of Culicidae and characterize their larval habitats in four Atlantic Forest areas of Northeastern Brazil.

During surveys conducted in the rainy seasons of August 2014, July to September 2021, and June to August 2022, we identified 18 Culicidae species inhabiting various larval habitats. Immatures were collected using dippers and pipettes from dams, flooded areas, natural and artificial containers, and even macrophytes within groundwater to capture associated immatures. The sampling locations encompassed four areas in enclaved wet forests (*Brejo de Altitude*) and Atlantic Forest fragments in Pernambuco state: i) the urban perimeter of Triunfo city (7°50’27.7”S, 38°06’20.5”W; 1100 m a.s.l); ii) the perimeter between the Chã de Alegria city and the Wildlife refuge Mata do Camucim (7°59’18.8”S, 35°13’39.0”W; 7°59’02.4”S, 35°12’07.2”W; 8°02’06.4”S, 35°10’39.8”W; 8°02’56.6”S, 35°12’44.7”W; 150-220 m a.s.l); iii) the perimeter of Dois Irmãos State Park and the campus of Rural Federal University of Pernambuco (8°00’29.5”S, 34°56’34.3”W; 8°00’06.0”S, 34°57’03.5”W; 8°01’20.6”S, 34°57’20.1”W; 8°01’12.4”S, 34°56’36.2”W; 5-30 m a.s.l); and iv) the Recife Botanical Garden (8°04’40.1”S, 34°57’59.9”W, 22 m a.s.l) (Table 1). 

To identify species, twenty specimens of each morphotype were isolated from each larval habitat and reared in plastic containers until adulthood. Upon reaching adulthood, the specimens were mounted on triangular boards. Fourth instar larval exuviae, pupae, and male genitalia were mounted on slides using Canada balsam or Entellan. Voucher specimens (Table 1) are deposited in the Entomological Collection Padre Jesus Santiago Moure at the Federal University of Paraná (DZUP, UFPR). Adult and immature mosquitoes were identified using established keys^9^ ^),(^ ^10^ ^),(^ ^11^ ^),(^ ^12^ ^),(^ ^13^, and the nomenclature and abbreviations followed existing guidelines^14^ ^),(^ ^15^.

**TABLE 1:** New records of Culicidae species collected as immature or adult mosquitoes in Pernambuco state, Brazil.

	Latitude	Longitude	Municipality	Larval Habitats	Registration date	Brazilian states	Voucher
Anophelinae							
*Anopheles* (*Stethomyia*) *kompi* Edwards, 1930	-8.0061	-34.9499	Recife	concrete water tank	07/22/2022	AC, AL, AM, BA, CE, DF, GO, PA, PB, PE, MT, MS, RJ, RR, SE, SP	DZUP 604049 to 604052
Culicinae							
Aedini							
*Georgecraigius* (*Horsfallius*) *fluviatilis* (Lutz, 1904)	-7.9900	-35.2075	Chã de Alegria	tire track	06/21/2022	AL, AM, BA, CE, ES, GO, MT, MG, PA, PB, PE, PR, RJ, RN, RS, SC, SE, SP, TO	DZUP 604053 to 604055
Culicini							
*Culex* (*Culex*) *bidens* Dyar, 1922	-8.0161	-34.9491	Recife	temporary pond	07/28/2021	AL, AM, GO, MG, MS, PA, PE, PR, RJ, RS, SP	DZUP 604062
	-8.0159	-34.9492			07/28/2021		DZUP 604063 - 604064
	-8.0165	-34.9488			07/28/2021		DZUP 604065
*Culex* (*Culex*) *chidesteri* Dyar, 1921	-8.0068	-35.2091	Chã de Alegria	temporary pond	08/16/2021	AM, ES, MG, MS, MT, PE, PR, RN, RJ, RS, SE, SP	DZUP 604066 - 604067
	-8.0264	-35.2043		temporary water reservoir	06/28/2022		DZUP 604068 - 604069
*Culex* (*Culex*) *mollis* Dyar & Knab, 1907	-8.0095	-34.9475	Recife	artificial water tank	09/29/2021	AM, GO, MG, MS, MT, PA, PE, PR, RJ, RO, RS, SP	DZUP 604070 to 604073
*Culex* (*Culex*) *usquatus* Dyar, 1918	-8.0400	-35.1944	São Lourenço da Mata	ditch	08/14/2021	AM, PA, PE, PR, RJ, RO, SP	DZUP 604077 to 604083
	-8.0282	-35.2022		tire track	06/20/2022		DZUP 604084 - 604085
	-8.0108	-35.2060		tire track	06/21/2022		DZUP 604086
	-8.0250	-35.2045		tire track	06/28/2022		DZUP 604087 - 604088
	-8.0373	-35.2000		temporary pond	06/18/2022		DZUP 604089
*Culex* (*Melanoconion*) *bastagarius* Dyar & Knab, 1906	-8.0332	-35.2008	São Lourenço da Mata	temporary water reservoir	08/14/2021	AM, BA, ES, GO, MG, MS, PA, PE, PR, RJ, RO, RS, SE, SP	DZUP 604090
	-8.0311	-35.2007			08/17/2021		DZUP 604091 - 604092
	-8.0211	-34.9544	Recife	temporary pond	08/16/2022		DZUP 604093
*Culex* (*Melanoconion*) *dunni* Dyar, 1918	-8.0114	-34.9466	Recife	flooded area	07/20/2022	AC, AM, MG, MS, PA, PE, PR, SP	DZUP 604094
*Culex* (*Melanoconion*) *serratimarge* Root, 1927	-8.0367	-35.1901	São Lourenço da Mata	flooded area	06/15/2022	AM, PE, RO, SP	DZUP 604095 - 604096
	-8.0348	-35.1860		flooded area	06/15/2022		DZUP 604097 - 604098
	-8.0401	-35.1968		temporary pond	06/16/2022		DZUP 604099 - 604100
*Culex* (*Melanoconion*) *ybarmis* Dyar, 1920	-8.0389	-35.1919	São Lourenço da Mata	ditch	06/15/2022	AM, PA, PE	DZUP 604101 to 604104
	-8.0401	-35.1944		ditch	06/15/2022		DZUP 604105 - 604106
	-8.0401	-35.1968		temporary pond	06/16/2022		DZUP 604107
*Culex* (*Microculex*) *imitator* Theobald, 1903	-8.0090	-34.9480	Recife	bromeliad	09/27/2021	AM, BA, ES, GO, MG, MT, PE, PR, RJ, RS, SC, SP	DZUP 604108
	-8.0099	-34.9475	Recife		09/27/2021		DZUP 604109 to 604112
	-8.0424	-35.2075	Vitória de Santo Antão		06/16/2022		DZUP 604113 - 604114
*Culex* (*Microculex*) *microphyllus* Root, 1927	-7.8410	-38.1056	Triunfo	bromeliad	08/23/2014	PE, PR, RJ, SP	BR09_14
	-8.0398	-35.1996	São Lourenço da Mata		08/15/2021		DZUP 604115
	-8.0099	-34.9475	Recife		09/27/2021		DZUP 604116
	-8.0109	-34.9468	Recife		09/27/2021		DZUP 604117
	-8.0130	-34.9450	Recife		09/29/2021		DZUP 604118
Mansoniini							
*Mansonia* (*Mansonia*) *humeralis* Dyar & Knab, 1916	-8.0420	-35.1962	São Lourenço da Mata	permanent water reservoir	06/14/2022	AM, BA, CE, MA, MG, MS, MT, PA, PB, PE, PR, RJ, RN, RO, RS, SP	DZUP 604119
Sabethini							
*Sabethes* (*Sabethes*) *purpureus* (Theobald, 1907)	-8.0778	-34.9666	Recife	---	08/08/2022	AM, GO, MG, MT, MS, PE, PR, RJ, RS, SC, SP, TO	DZUP 604135
*Wyeomyia* (*Phoniomyia*) *incaudata* Root, 1928	-8.0148	-34.9513	Recife	phytotelm	07/28/2021	BA, GO, MG, PE, PR, RJ, SC, SP	---
	-8.0146	-34.9475	Recife	bromeliad	09/23/2021		---
	-8.0099	-34.9475	Recife	bromeliad	09/27/2021		DZUP 604122 to 604124
	-8.0425	-35.2072	Vitória de Santo Antão	bromeliad	08/15/2021		DZUP 604121
*Wyeomyia* (*Phoniomyia*) *pilicauda* Root, 1928	-8.0424	-35.2073	Vitória de Santo Antão	bromeliad	06/16/2022	GO, PA, PE, PR, RJ, SC, SP	DZUP 604125
	-8.0424	-35.2075			06/16/2022		DZUP 604126
*Wyeomyia* (*Spilonympha*) *airosai* Lane & Cerqueira, 1942	-8.0425	-35.2072	Vitória de Santo Antão	bromeliad	08/15/2021	ES, PE, RJ, SP	DZUP 604127
Uranotaeniini							
*Uranotaenia* (*Uranotaenia*) *apicalis* Theobald, 1903	-8.0126	-35.2043	São Lourenço da Mata	permanent water reservoir	08/16/2021	AC, AM, MT, PE, PR, SE, SP, TO	DZUP 604128
	-8.0261	-35.2044		fish tank	06/28/2022		DZUP 604129

**DZUP:** Padre Jesus Santiago Moure Entomological Collection; **AC:** Acre; **AL:** Alagoas; **AM:** Amazonas, **BA:** Bahia; **CE:** Ceará; **DF:** Distrito Federal; **ES:** Espírito Santo; **GO:** Goiás; **MG:** Minas Gerais; **MT:** Mato Grosso; **MS:** Mato Grosso do Sul; **PA:** Pará; **PE:** Pernambuco; **PR:** Paraná; **RJ:** Rio de Janeiro; **RN:** Rio Grande do Norte; **RO:** Rondônia; RR: Roraima; **RS:** Rio Grande do Sul; **SC:** Santa Catarina; **SE:** Sergipe; **SP:** São Paulo; **TO:** Tocantins.

The newly recorded species belong to five tribes within the subfamily Culicinae: Aedini, Culicini, Mansoniini, Sabethini, and Uranotaeniini. Additionally, one species belongs to the subfamily Anophelinae. Immatures of *Georgecraigius* (*Horsfallius*) *fluviatilis* (Lutz, 1904) (Figure 1A), a member of the Aedini tribe, were discovered in a temporary tire track excavated in clayey soil near the urban center of Chã de Alegria. The larval habitat lacked shading and had a temperature of 29.3°C, a pH of 7.8, a conductivity of 200 µS/cm, and total solids of 90 mg/L. 

**FIGURE 1:**
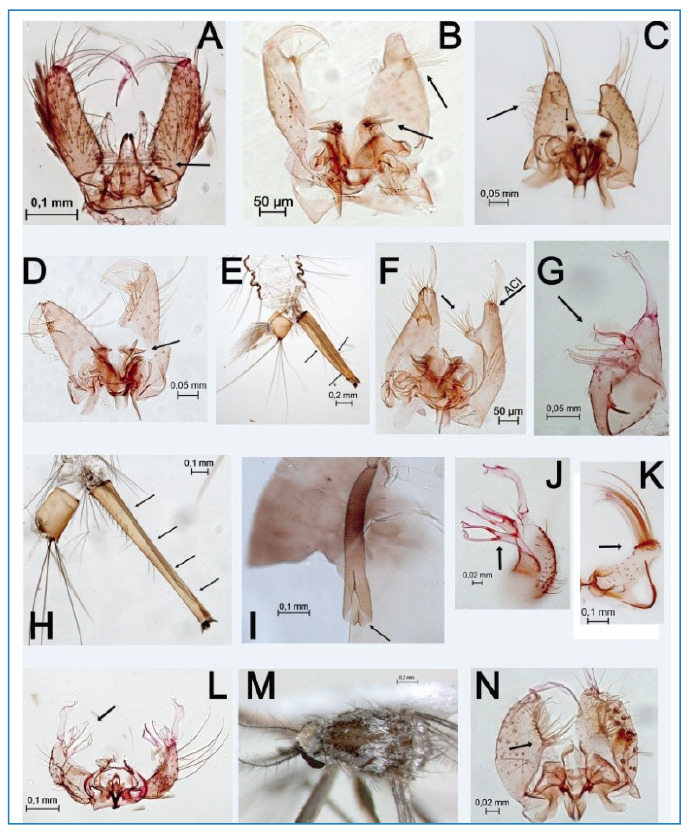
Species plate. **(A)** Male genitalia of *Georgecraigius* (*Horsfallius*) *fluviatilis*. The gonocoxite bears internally basal thick setae (seta) and an elongate claspette, thickened before apex; **(B)** Male genitalia of *Culex* (*Culex*) *bidens*. The seta points to the undivided apical lobe and the ventral arm of the mesosome with three strong teeth. **(C)** Male genitalia of *Culex* (*Culex*) *chidesteri*. The seta points to the undivided apical lobe. The proctiger bears pointed spines apically and spatulate spines laterally (seta). (D, E) *Culex* (*Culex*) *mollis*. **(D)** Male genitalia with ventral arm of mesosome T-shaped (seta). **(E)** The general appearance of larval abdominal segments VIII-X. The siphon has two double long tufts and a single apical one (setae). **(F)** Male genitalia of *Culex* (*Culex*) *usquatus.* The apical cluster of setae has a long setae and an apical lobe with 12-15 subequal, grouped, gently curved setae (seta). **(G)** Male genitalia of *Culex* (*Melanoconion*) *bastagarius.* The subapical lobe is divided. The proximal division has two long and hooked setae (one swollen near the tip) (seta). The distal division is subdivided. **(H, I)** *Culex* (*Melanoconion*) *dunni*. **(H)** The general appearance of larval abdominal segments VIII-X. The siphon has four visible dorsal pairs (setae) and three ventral pairs. **(I)** Pupa trumpet widens distally with a large emargination on the distal margin opposite the meatal cleft (seta). **(J, K)** *Culex* (*Melanoconion*) *serratimarge*. **(J)** Male genitalia. The subapical lobe is divided. The proximal division has two long and hooked setae, one swollen near the tip (seta). The distal division is subdivided into two short arms. **(K)** Lobes of tergum IX are large with broad, lateral prolongations bearing several rows of long hairs (seta) **(L)** Male genitalia of *Culex* (*Melanoconion*) *ybarmis*. The subapical lobe is divided. The distal division bears long-curved setae, including a slender pointed one, a small leaf, and three foliaceous setae (seta) **(M, N)** *Culex* (*Microculex*) *imitator*. **(M)** Detail of the adult male mesonotum with silver scales on the mid-anterior portion, extending laterally to the prescutellar area. **(N)** Male genitalia with a divided subapical lobe. The proximal division has two long, strong, tip-curved setae (seta).

Ten species belonging to the Culicini tribe were recorded. Immatures of *Culex* (*Culex*) *bidens* Dyar, 1922 (Figure 1B) were found in diverse temporary puddles located within open or partially shaded vegetation areas in the urban zone of Recife. These puddles contained submerged marginal grasses and were closely spaced, ranging from 25 to 50 meters apart. The abiotic water conditions varied, with temperatures ranging from 28.8°C to 30°C and pH between 6.9 and 7.2. 

*Culex* (*Culex*) *chidesteri* Dyar, 1921 (Figure 1C) was found in both temporary puddles and permanent water reservoirs. The former were located in agricultural areas, while the latter were found in rural areas. Both larval habitats were characterized by substantial submerged marginal grasses and direct exposure to sunlight. Notably, both environments were used for local bovine and swine watering. The abiotic conditions in these habitats varied, with temperatures ranging from 27.8°C and 28.7°C, pH between 6.6 and 7, conductivity between 80 and 480 µS/cm, and total dissolved solids between 30 and 230 mg/L.

Immatures of *Culex* (*Culex*) *mollis* Dyar & Knab, 1906 (Figure 1D, 1E) were found exclusively in an artificial water tank used for domestic water supply. This completely shaded larval habitat was situated near the forested area of Dois Irmãos State Park. A single larva of *Toxorhynchites* was also found in the same habitat. At the time of collection, the abiotic conditions were: temperature of 27.5°C, pH 6.5, conductivity of 110 µS/cm, and total solids of 50 mg/L. Notably, to our knowledge, this is the first record of this species in northeastern Brazil. 

An abundance of *Culex* (*Culex*) *usquatus* Dyar, 1918 (Figure 1F) larvae were discovered in puddles formed by tire tracks and shallow rainfall runoff ditches in rural and agricultural areas. These open, unshaded larval habitats contained muddy water. The abiotic conditions varied: temperature from 26°C to 33.2°C, pH levels between 6.5 and 7.7, conductivity 50 to 430 µS/cm, and total solids 30 to 210 mg/L. Interestingly, larvae of *Cx. maxi*, *Cx. nigripalpus* Theobald, 1901, *Cx. ybarmis* Dyar, 1920, *Ochlerotatus scapularis* (Rondani, 1848), *Psorophora ferox* (Humboldt, 1819), and *Uranotaenia lowii* Theobald, 1901 were also found in the same larval habitat. Notably, to our knowledge, this is the first record of this species in northeastern Brazil. 

Immatures of *Culex* (*Melanoconion*) *bastagarius* Dyar & Knab, 1906 (Figure 1G) were primarily found in temporary reservoirs used for watering animals in rural areas. In Recife, they were found in a temporary puddle within an open vegetation area near the UFRPE experimental livestock station. Interestingly, in all cases, immature mosquitoes developed in close proximity to cattle pens and human settlements. In rural areas, these reservoirs are sometimes visited by the board-snouted *Caiman (Caiman) latirostris* (Daudin, 1802), as confirmed by researchers and residents. The larval habitats were characterized by abundant marginal grasses and variable abiotic conditions: temperature between 28.7°C and 31.4°C, pH between 6.5 and 7.7, conductivity between 120 and 480 µS/cm, and total dissolved solids between 60 and 230 mg/L. Notably, larvae of *Anopheles* (*Nyssorhynchus*) *triannulatus* (Neiva & Pinto, 1922) were also identified in the same larval habitat.

Immatures of *Culex* (*Melanoconion*) *dunni* Dyar, 1918 (Figure 1H,I) were exclusively found in a permanently flooded area adjacent to the Dois Irmãos State Park Zoo (8°00'41.2"S, 34°56'47.8"W). The sampling area was completely shaded and contained significant amounts of floating and emergent macrophytes, filamentous algae, and decaying plant material. Furthermore, solid waste generated by improper garbage disposal by park visitors was observed. The abiotic conditions at the time of sampling were: temperature of 26.9°C, pH 6.1, conductivity of 90 µS/cm, and total dissolved solids of 30 mg/L. To our knowledge, this is the first record of this species in northeastern Brazil. 

Two species belonging to the *Melanoconion* subgenus were identified: *Culex* (*Melanoconion*) *serratimarge* Root, 1927 (Figure 1J,K) and *Culex* (*Melanoconion*) *ybarmis* Dyar, 1920 (Figure 1L). Both species were primarily found in temporary puddles and flooded areas formed during rainwater runoff. *Culex* (*Mel.*) *serratimarge* preferred shaded habitats rich in decaying organic matter, while *Cx.* (*Mel.*) *ybarmis* favored open vegetation areas with partially submerged plants. The abiotic conditions for both species ranged from 24.7°C in shaded areas to 27.6°C in more exposed environments, with a pH range of 5.9 to 7.2 and conductivity of 80 to 210 µS/cm. Total dissolved solids ranged from 30 to 100 mg/L. Notably, to our knowledge, this study represents the first record of both species in northeastern Brazil. 

Only two species belonging to the *Microculex* subgenus were recorded: *Culex* (*Microculex*) *imitator* Theobald, 1903 (Figure 1M,N) and *Culex* (*Microculex*) *microphyllus* Root, 1927 (Figure 2A,B). Immatures of both species were found within the bromeliads *Aechmea leptantha* (Harms) Leme and J.A. Siqueira, often sharing the same larval habitat in both forested and urban areas. These partially open or shaded bromeliad habitats contained substantial amounts of decomposing organic matter. The abiotic conditions recorded were: temperature range of 26.5°C to 30.2°C, pH range of 4 to 5.9, conductivity range of 60 to 570 µS/cm, and total dissolved solids range of 20 and 300 mg/L. This study marks the first record of *Cx. microphyllus* in the northeast region, and it was one of the most frequently encountered species in bromeliads found at the interface with urban areas. 

**FIGURE 2:**
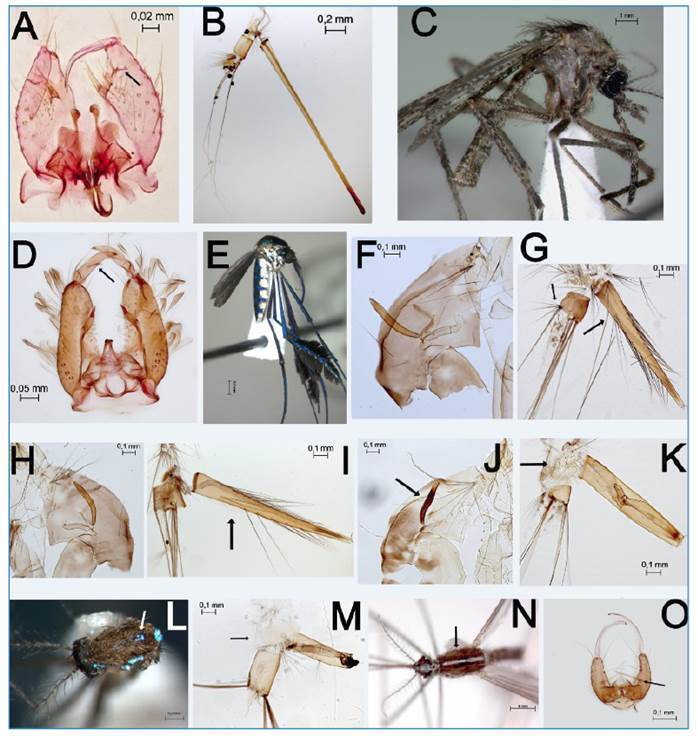
Species plates. (A, B) *Culex* (*Microculex*) *microphyllus*. **(A)** Male genitalia: The subapical lobe is divided, with the distal division bearing a small leaf (seta) **(B)** General appearance of the larval abdominal segments VIII-X: The siphon is very long and slender **(C, D)** *Mansonia* (*Mansonia*) *humeralis*. **(C)** The general appearance of adult females. **(D)** Male genitalia: The gonostylus has protuberances on the middle internal margin. **(E)** *Sabethes* (*Sabethes*) *purpureus*: General appearance of the adult female. **(F, G)** *Wyeomyia* (*Phoniomyia*) *incaudata*. **(F)** Pupa trumpet: The trumpet is cylindrical and slightly curved. **(G)** The general appearance of the larval abdominal segments VIII-X: The false pecten of the siphon has 10 spines (seta), and seta 4-X has seven branches (seta). **(H, I)** *Wyeomyia* (*Phoniomyia*) *pilicauda.* **(H)** Pupa trumpet: The trumpet is long, cylindrical, and curved. **(I)** The general appearance of the larval abdominal segments VIII-X: The false pectens have more than 15 spines (setae). **(J, K)** *Wyeomyia* (*Spilonympha*) *airosai*. **(J)** The pupal trumpet is strongly sclerotized and broadened medially (seta). **(K)** The general appearance of the larval abdominal segments VIII-X: The pecten of segment VIII has six long spines and several smaller lateral spines inserted into a sclerotized plate (seta). The siphons are densely covered with small tufts. **(L, M)** *Uranotaenia* (*Uranotaenia*) *apicalis*. **(L)** The mesonotum has brown and iridescent scales on the antepronotum, paratergite, middle lobe of the scutellum, and a single spot above the prescutellar area (seta). **(M)** The general appearance of the larval abdominal segments VIII-X: The pecten of segment VIII has seven strong spines inserted into a large sclerotized plate (seta). **(N, O)** *Anopheles* (*Stethomyia*) *kompi*. **(N)** The mesonotum has a brown integument and a silvery stripe extending from the cervical sclerites to the scutellum (seta). **(D)** Male genitalia with an accessory seta inserted in the proximal third of the gonocoxite (seta).

Immatures of *Mansonia* (*Mansonia*) *humeralis* Dyar & Knab, 1916 (Figure 2C,D) were found exclusively in the Tapacurá Reservoir, associated with *Eichhornia crassipes* macrophytes. This larval habitat presented average abiotic conditions of 28.9°C, a pH of 7.1, a conductivity of 415 µS/cm, and total dissolved solids of 195 mg/L. Notably, the sampling period (June 2022) coincided with the peak water level in the reservoir and a high abundance of both *M. humeralis* and *Mansonia* (*Mansonia*) *wilsoni* (Barreto & Coutinho, 1944), which was also present in the same habitat. 

Four Sabethini species were identified. *Sabethes* (*Sabethes*) *purpureus* (Theobald, 1907) (Figure 2E) was the only species collected as adults in the Botanical Garden of Recife (8°04'40.1"S, 34°57'59.9"W), a small (11 ha) Atlantic Forest park within the urban area. Their presence near human settlements warrants attention due to their potential vector roles. This represents the first record of this species in northeastern Brazil. The remaining three species, *Wyeomyia* (*Phoniomyia*) *incaudata* Root, 1928 (Figure 2F,G), *Wyeomyia* (*Phoniomyia*) *pilicauda* Root, 1928 (Figure 2H,I), and *Wyeomyia* (*Spilonympha*) *airosai* Lane & Cerqueira, 1942 (Figure 2J,K) were found exclusively associated with the bromeliad *Aechmea leptantha*. 

*Wyeomyia* (*Pho.*) *incaudata* was found in bromeliads used for ornamental purposes in urban areas, as well as in native bromeliads. Meanwhile, *Wy.* (*Pho.*) *pilicauda* and *Wy.* (*Spi.*) *airosai* were found exclusively in forested areas. These mosquitoes often shared habitats with *Wyeomyia* (*Wyeomyia*) *medioalbipes* Lutz, 1904; *Cx. imitator* and *Cx. microphyllus*. The abiotic conditions of these larval habitats varied: temperature 26.5°C to 30°C, pH 4.7 to 6.9, conductivity 0 to 140 µS/cm, and total dissolved solids 0 to 60 mg/L. Except for *Wy. incaudata*, all these species were recorded for the first time in northeastern Brazil. 

*Uranotaenia* (*Uranotaenia*) *apicalis* Theobald, 1903 (Figure 2J,M) was found in artificial larval habitats, including a densely populated fish tank and a permanent reservoir, both containing the macrophyte *Pistia stratiotes* L. Larvae of *Aedeomyia* (*Aedeomyia*) *squamipennis* (Lynch Arribálzaga, 1878) and *An.* (*Nys.*) *triannulatus* was also observed in the same habitat. Abiotic conditions varied: temperature 26.7°C to 30°C, pH 7.3 to 7.5, conductivity 290 to 520 µS/cm, and total dissolved solids 140 to 250 mg/L. This study represents one of the first records of *Uranotaenia apicalis* development in fish tanks. 

For the Anophelinae subfamily, we recorded a species of the subgenus *Stethomyia* in Pernambuco for the first time. *Anopheles* (*Stethomyia*) *kompi* Edwards, 1930 (Figure 2M,O) was collected from a partially shaded concrete water tank located near a large urban supply reservoir. The tank contained submerged plants and decaying leaves, and the water was transparent with a temperature of 26.7°C and pH of 4.3 at the time of collection. Future surveys should carefully evaluate the presence of *An. kompi* near houses and artificial larval habitats, particularly alongside *Cx. mollis* and *Cx. bastagarius*.

This study significantly expands our understanding of mosquito diversity in Brazil. We identified nine species for the first time in northeastern Brazil and 18 species for the first time in Pernambuco, bringing the total number of recorded species in the state to 94 ()(Supplementary Material Table S1). Additionally, our study provides valuable insights into the biology of immature mosquito larvae and the environmental conditions of their larval habitats. While some identified species are potential vectors of pathogens and parasites affecting humans and wildlife, they have not yet been linked to disease transmission cycles in this region. This highlights the importance of studies that assess immature mosquito communities, as they offer new information on the geographic distribution and ecological aspects of the species. Further surveys of mosquito populations across different biomes and environmental impact levels are crucial for assessing potential risks to human and animal health in Brazil.
