# Supplementary material for: New occurrences of mosquitoes (Diptera: Culicidae) in the Atlantic Forest biome of the Brazilian Northeast
Source: Rev Soc Bras Med Trop. 2024 Feb 5;57:e00701-2024. doi: 10.1590/0037-8682-0513-2024 (PMC10852454; doi:10.1590/0037-8682-0513-2024)
Supplement: Supplementary file 1 [file 1678-9849-rsbmt-57-e00701-2024-supp1.pdf]

Supplementary Table S1: First records of Culicidae species collected as immature or adult mosquitoes in Pernambuco state, Brazil.

	Latitude	Long	Municipality	Larval Habitats	Registration date	Brazilian states	Voucher	References list
Anophelinae								
Anopheles (*Anopheles*) *eiseni* Coquillett, 1902	---	---	---	---	---	AM, MT, PA, PE, PR, RJ, RO, SP	---	3
Anopheles (*Anopheles*) *fluminensis* Root, 1927	---	---	---	---	---	AC, GO, MG, MS, MT, PA, PE, PR, RJ, RS, SC, SP	---	3
Anopheles (*Anopheles*) *medialis* Harbach, 2018	---	---	---	---	1939 1944	PE, RR, SP	---	6 (as *Anopheles intermedius*)
Anopheles (*Anopheles*) *minor* da Costa Lima, 1929	---	---	---	---	1939 1944	AP, BA, ES, MT, PA, RO, SE	---	6
Anopheles (*Anopheles*) *peryassui* Dyar & Knab, 1908	---	---	---	---	1939 1944	AC, AM, AP, BA, MG, MS, MT, PA, PE, RJ, RO, RR, TO	---	6
	---	---	Recife	---	09/21/1938		CMN 19083	Preserved at Fiocruz-CMN
Anopheles (*Kerteszia*) *bellator* Dyar & Knab, 1906	---	---	---	---	---	BA, PE, PR, SP	---	3
Anopheles (*Kerteszia*) *cruzii* Dyar & Knab, 1908	---	---	---	---	---	ES, GO, MG, MT, PE, PR, RJ, RS, SC, SP	---	3
Anopheles (*Kerteszia*) *homunculus* Komp, 1937	---	---	Recife	---	8/10/1934	BA, ES, PE, PR, RJ, RS, SC, SP	CMN 2358	Preserved at Fiocruz-CMN
Anopheles (*Nyssorhynchus*) *albimanus* Wiedemann, 1820	-8.161	-34.927	Recife	---	08/25/1944	AC, AL, BA, ES, GO, MG, PA, PB, PE, PR, RJ, RN, SE, SP	FMNHINS 0004 101 269	Preserved at FMNH
	---	---	Ipojuca	---	08/28/1954		CMN 43566	Preserved at Fiocruz-CMN
Anopheles (*Nyssorhynchus*) *albitarsis* Lynch Arribálzaga, 1879	---	---	Recife	---	1/10/1929	AC, BA, CE, AM, AP, ES, GO, MA, MG, MS, MT, PA, PE, PR, RJ, RN, RO, RR, RS, SE, SP, TO	s/n	Preserved at Fiocruz-CMN
	---	---	Petrolina	---	4/3/1933		CMN 551	
	---	---	---	---	1939-1944		---	6
	---	---	Cabrobó	---	1/7/1947		s/n	Preserved at Fiocruz-CMN
Anopheles (*Nyssorhynchus*) *aquasalis* Curry, 1932	---	---	Gloria do Goitá	---	4/24/1905	AC, AM, AP, CE, ES, GO, MA, MS, PA, PB, PE, RJ, RN, SP,	---	6
Anopheles (*Nyssorhynchus*) *argyritarsis* Robineau-Desvoidy, 1827	---	---	Recife	---	4/12/1905	AC, AM, BA, CE, DF, ES, GO, MA, MG, MS, MT, PB, PE, PI, PR, RJ, RR, RS, SC, SE, SP, TO,	s/n	Preserved at Fiocruz-CMN
	---	---	---	---	1939-1944		---	6
	---	---	Recife	---	06/19/1942		CMN 27040	Preserved at Fiocruz-CMN

Continue...

Supplementary Table S1: Continuation.

	Latitude	Long	Municipality	Larval Habitats	Registration date	Brazilian states	Voucher	References list
Anopheles (*Nyssorhynchus*) *braziliensis* (Chagas, 1907)	---	---	Recife	---	4/12/1905	AC, AM, AP, GO, MA, MG, MS, MT, PA, PE, PR, RO, RN, RR, SP	s/n	Preserved at Fiocruz-CMN
	---	---	Recife	---	06/19/1942		CMN 27040	
	---	---	Ipojuca	---	08/28/1954		CMN 43565	
	---	---	---	---	1939-1944		---	6 (as *Anopheles pessoai*)
Anopheles (*Nyssorhynchus*) *darlingi* Root, 1927	---	---	Belém do São Francisco	---	07/21/1947	AC, AM, AP, BA, ES, GO, MA, MG, MS, MT, PA, PE, PR, RJ, RO, RR, SC, SP, TO	s/n	Preserved at Fiocruz-CMN
	---	---	---	---	---		---	3
Anopheles (*Nyssorhynchus*) *evansae* (Brèthes, 1926)	---	---	Recife	---	1/10/1924	BA, ES, GO, MA, MG, MS, MT, PI, PR, RJ, RO, RR, RS, SC, SP, TO,	s/n	Preserved at Fiocruz-CMN
	---	---	Recife	---	1/10/1939		s/n	
	---	---	---	---	1939-1944		---	6 (as *Anopheles noroestensis*)
Anopheles (*Nyssorhynchus*) *lutzii* (Theobald, 1901)	---	---	---	---	---	ES, GO, MG, PE, PR, RJ, MT, RS, SC, SP	---	3
Anopheles (*Nyssorhynchus*) *oswaldoi* (Peryassú, 1922)	---	---	Recife	---	1/10/1924	AC, AM, AP, BA, ES, GO, PI, MA, MG, MS, MT, PE, PR, RJ, RO, RR, RS, SE, SP, TO	s/n	Preserved at Fiocruz-CMN
	---	---	Limoeiro	---	8/9/1934		CMN 2224	Preserved at Fiocruz-CMN
Anopheles (*Nyssorhynchus*) *parvus* (Chagas, 1907)	---	---	---	---	---	DF, ES, GO, MG, MS, MT, PE, PR, RJ, SC, SP, TO	---	3
Anopheles (*Nyssorhynchus*) *strodei* Root, 1926	---	---	---	---	---	AC, AM, BA, ES, GO, MA, MG, MS, MT, PA, PE, PR, RJ, RO, RR, RS, SC, SP, TO	---	3
	---	---	Ipojuca	---	08/28/1954		CMN 43566	Preserved at Fiocruz-CMN
Anopheles (*Nyssorhynchus*) *triannulatus* (Neiva & Pinto, 1922)	-8.050	-34.900	Recife	---	09_1929	AC, AM, AP, BA, ES, GO, MA, MG, MS, MT, PA, PB, PE, PR, RJ, RN, RO, RR, SE, SP, TO,	BRNC255 - 9075305	Preserved at USNM
Anopheles (*Stethomyia*) *kompi* Edwards, 1930	-8.006	-34.950	Recife	concrete water tank	07/22/2022	AC, AL, AM, BA, CE, DF, GO, PA, PB, PE, MS, MT, RJ, RR, SE, SP	DZUP 604049 to 604052	present study
Culicinae								
Aedeomyiini								

Continue...

Supplementary Table S1: Continuation.

	Latitude	Long	Municipality	Larval Habitats	Registration date	Brazilian states	Voucher	References list
Aedeomyia (Aedeomyia) squamipennis (Lynch Arribálzaga, 1878)	---	---	Recife	---	1/10/1929	AC, AM, AP, BA, CE, GO, MA, MG, MS, MT, PA, PE, PR, RJ, RN, RO, RS, SC, SE, SP, TO	s/n	Preserved at Fiocruz-CMN
	---	---		---	08/27/1942		CMN 27045	Preserved at Fiocruz-CMN
Aedini								
Georgecraigius (Horsfallius) fluviatilis (Lutz, 1904)	-7.990	-35.208	Chã de Alegria	tire track	06/21/2022	AL, AM, BA, CE, ES, GO, MT, MG, PA, PB, PE, PR, RJ, RN, RS, SC, SE, SP, TO	DZUP 604053 to 604055	present study
Haemagogus (Conopostegus) leucocelaenus (Dyar & Shannon, 1924)	---	---	Recife	---	07/23/1948	AC, AM, AP, BA, CE, DF, ES, GO, MA, MG, MS, MT, PA, PE, PR, RJ, RN, RS, SC, SE, SP, TO	CMN 42811	Preserved at Fiocruz-CMN
Haemagogus (Haemagogus) capricornii Lutz, 1904	---	---	São Gonçalo, Petrolina	---	4/15/1905	ES, GO, MG, MS, PA, PE, PR, RJ, SP	CMN 206	Preserved at Fiocruz-CMN
Haemagogus (Haemagogus) janthinomys Dyar, 1921	---	---	Recife	---	07/20/1948	AC, AM, AP, BA, CE, DF, ES, GO, MA, MG, MS, MT, PA, PB, PE, PR, RJ, RO, RR, SP, TO	CMN 42799-1	Preserved at Fiocruz-CMN
	---	---	Recife	---	07/2009-11/2009		---	3
Haemagogus (Haemagogus) spegazzinii Brèthes, 1912	---	---	Petrolina	---	03_1933	AC, AM, AP, BA, CE, ES, GO, MA, MG, MS, MT, PA, PB, PE, PI, PR, RJ, RN, SE, SP, TO	CMN 550	Preserved at Fiocruz-CMN
	---	---	Recife	---	01/29/1946		CMN 41379	Preserved at Fiocruz-CMN
Howardina fulvithorax (Lutz, 1904)	---	---	Recife	---	01/29/1946	AC, AM, BA, CE, ES, GO, MG, MS, MT, PA, PE, PI, PR, RJ, SE, SP, TO	CMN 41379	Preserved as Aedes fulvithorax at Fiocruz-CMN
Ochlerotatus lepidus (Cerqueira & Paraense, 1945)	---	---	Águas belas	---	03/26/1954	AL, CE, MG, PB, PE, RN	CMN 43478	Preserved as Aedes lepidus at Fiocruz-CMN
	---	---	Inajá	---	07/29/1954		CMN 43553	
	---	---	Serrita	---	---		CMN 43522	
	---	---	Belo Jardim	---	07/22/1955		CMN 43600	
Ochlerotatus (Culicelsa) taeniorhynchus (Wiedemann, 1821)	---	---	Recife	---	11/1/1946	BA, CE, DF, ES, MA, MG, PA, PB, PE, RJ, RN, SE, SP	CMN 41686	Preserved as Aedes taeniorhynchus at Fiocruz-CMN

Continue...

Supplementary Table S1: Continuation.

	Latitude	Long	Municipality	Larval Habitats	Registration date	Brazilian states	Voucher	References list
Ochlerotatus (Ochlerotatus) scapularis (Rondani, 1848)	---	---	Recife	---	4/12/1905	AC, AM, AP, BA, CE, DF, ES, MA, MG, MS, MT, PA, PB, PE, PR, RJ, RN, RS, SC, SE, SP, TO	s/n	Preserved as Aedes scapularis at Fiocruz-CMN
	---	---	Petrolina	---	4/3/1933		CMN 551	
	---	---	Recife	---	01/29/1946		CMN 41379	
Ochlerotatus (*Protoculex*) *serratus* (Theobald, 1901)	---	---	Recife	---	07/20/1948	AC, AM, AP, BA, DF, ES, GO, MA, MG, MS, MT, PA, PE, PR, RJ, RN, RO, RS, SC, SE, SP, TO	CMN 42799	Preserved as Aedes serratus Fiocruz-CMN
'*Ochlerotatus (Protomacleya)*'' *argyrothorax* (Bonne-Wepster & Bonne, 1920)	---	---	Recife	---	08/20/1948	AM, AP, BA, DF, ES, MG, MS, MT, PA, PE, PR, RJ, RO, SE, SP	CMN 42799	Preserved as Aedes argyrothorax at Fiocruz-CMN
Psorophora (*Janthinosoma*) *ferox* (von Humboldt, 1819)	---	---	Recife	---	before 1900	AC, AM, AP, BA, CE, DF, ES, GO, MA, MG, MS, MT, PA, PB, PE, PI, PR, RJ, RN, RO, RS, SC, SE, SP, TO	---	1 (as Janthinosoma musica)
	---	---		---	7/20/1948		CMN 42799	Preserved at Fiocruz-CMN
Psorophora (*Psorophora*) *ciliata* (Fabricius, 1794)	---	---	Recife	---	06_1930	AM, BA, CE, ES, GO, MG, MS, MT, PE, PR, RJ, RN, RS, SC, SP	s/n	Preserved at Fiocruz-CMN
Psorophora (*Psorophora*) *lineata* (von Humboldt, 1819)	---	---	Recife	---	4/13/1905	AC, AM, AP, PE	CMN 486	Preserved at Fiocruz-CMN
Stegomyia (*Stegomyia*) *aegypti* (Linnaeus, 1762)	---	---	Recife; Gravatá	---	before 1900	All states	---	1 (as *Stegomyia fasciata*)
	---	---	Timbaúba	---	07/1938 - 11/1938		CMN 19149	Preserved at Fiocruz-CMN
	---	---	Sertania	---	7/10/1946		CMN 41878	Preserved at Fiocruz-CMN
Stegomyia albopicta (Skuse, 1895)	---	---	Recife	---	9/1/1999	All states	---	2
Culicini								
Culex (*Culex*) *bidens* Dyar, 1922	-8.016	-34.949	Recife	temporary pond	7/28/2021	AL, AM, GO, MG, MS, PA, PE, PR, RJ, RS, SP	DZUP 604062	present study
	-8.016	-34.949			7/28/2021		DZUP 604063 - 604064	
	-8.017	-34.949			7/28/2021		DZUP 604065	

Continue...

Supplementary Table S1: Continuation.

	Latitude	Long	Municipality	Larval Habitats	Registration date	Brazilian states	Voucher	References list
Culex (*Culex*) *chidesteri* Dyar, 1921	-8.007	-35.209	Chã de Alegria	temporary pond	8/16/2021	AM, ES, MG, MS, MT, PE, PR, RN, RJ, RS, SE, SP	DZUP 604066 - 604067	present study
	-8.026	-35.204		temporary water reservoir	6/28/2022		DZUP 604068 - 604069	
Culex (*Culex*) *maxi* Dyar, 1928	-8.601	-38.569	Floresta	animal watering	6/1/2010	BA, MS, PE, RS, SE	---	4
Culex (*Culex*) *mollis* Dyar & Knab, 1907	-8.010	-34.948	Recife	artificial water tank	9/29/2021	AM, GO, MG, MS, MT, PA, PE, PR, RJ, RO, RS, SP	DZUP 604070 to 604073	present study
Culex (*Culex*) *nigripalpus* Theobald, 1901	-8.140	-35.084	Moreno	---	02/2008-03/2008	AC, AM, BA, GO, MG, MS, MT, PE, PR, RJ, RS, SP, TO	---	5
Culex (*Culex*) *quinquefasciatus* Say, 1823	---	---	Recife; Gravatá	---	before 1900	AC, AM, AP, BA, DF, ES, GO, MA, MG, MS, MT, PA, PE, PR, RJ, RN, RO, RS, SC, SE, SP	---	1 (as *Culex fatigans*)
	---	---	Recife	---	4/4/1932		CEIOC 22979	Preserved at Fiocruz-CEIOC
Culex (*Culex*) *usquatus* Dyar, 1918	-8.040	-35.194	São Lourenço da Mata	ditch	8/14/2021	AM, PA, PE, PR, RJ, RO, SP	DZUP 604077 to 604083	present study
	-8.028	-35.202		tire track	6/20/2022		DZUP 604084 - 604085	present study
	-8.011	-35.206		tire track	6/21/2022		DZUP 604086	present study
	-8.025	-35.205		tire track	6/28/2022		DZUP 604087 - 604088	present study
	-8.037	-35.200		temporary pond	6/18/2022		DZUP 604089	present study
Culex (*Melanoconion*) *bastagarius* Dyar & Knab, 1906	-8.033	-35.201	São Lourenço da Mata	temporary water reservoir	8/14/2021	AM, BA, ES, GO, MG, MS, PA, PE, PR, RJ, RO, RS, SE, SP	DZUP 604090	present study
	-8.031	-35.201		temporary water reservoir	8/17/2021		DZUP 604091 - 604092	present study
	-8.021	-34.954	Recife	temporary pond	8/16/2022		DZUP 604093	present study
Culex (*Melanoconion*) *dunni* Dyar, 1918	-8.011	-34.947	Recife	flooded area	7/20/2022	AC, AM, MG, MS, PA, PE, PR, SP	DZUP 604094	present study
Culex (*Melanoconion*) *ocossa* Dyar & Knab, 1919	-8.140	-35.084	Moreno	---	02/2008-03/2008	AM, MS, PA, PE, RJ, SP	---	5
Culex (*Melanoconion*) *serratimarge* Root, 1927	-8.037	-35.190	São Lourenço da Mata	flooded area	6/15/2022	AM, PE, RO, SP	DZUP 604095 - 604096	present study
	-8.035	-35.186		flooded area	6/15/2022		DZUP 604097 - 604098	present study
	-8.040	-35.197		temporary pond	6/16/2022		DZUP 604099 - 604100	present study

Continue...

Supplementary Table S1: Continuation.

	Latitude	Long	Municipality	Larval Habitats	Registration date	Brazilian states	Voucher	References list
Culex (*Melanoconion*) *spissipes* (Theobald, 1903)	-8.140	-35.084	Moreno	---	02/2008-03/2008	AC, AM, BA, MA, MT, PA, PE, PR, RJ, SP	---	5
Culex (*Melanoconion*) *ybarmis* Dyar, 1920	-8.039	-35.192	São Lourenço da Mata	ditch	6/15/2022	AM, PA, PE	DZUP 604101 to 604104	present study
	-8.040	-35.194		ditch	6/15/2022		DZUP 604105 - 604106	present study
	-8.040	-35.197		temporary pond	6/16/2022		DZUP 604107	present study
Culex (*Microculex*) *imitator* Theobald, 1903	-8.009	-34.948	Recife	bromeliad	9/27/2021	AM, BA, ES, GO, MG, MT, PE, PR, RJ, RS, SC, SP	DZUP 604108	present study
	-8.010	-34.948	Recife		9/27/2021		DZUP 604109 to 604112	present study
	-8.042	-35.208	Vitória de Santo Antão		6/16/2022		DZUP 604113 - 604114	present study
Culex (*Microculex*) *microphyllus* Root, 1927	-7.841	-38.106	Triunfo	bromeliad	8/23/2014	PE, PR, RJ, SP	BR09_14	present study
	-8.040	-35.200	São Lourenço da Mata		8/15/2021		DZUP 604115	
	-8.010	-34.948	Recife		9/27/2021		DZUP 604116	
	-8.011	-34.947	Recife		9/27/2021		DZUP 604117	
	-8.013	-34.945	Recife		9/29/2021		DZUP 604118	
Culex (*Microculex*) *pleuristriatus* Theobald, 1903	-8.140	-35.084	Moreno	---	02/2008-03/2008	BA, MG, PA, PE, RJ, RS, SC, SP	---	5
Mansoniini								
Coquillettidia (*Rhynchotaenia*) *albicosta* (Chagas, 1908)	---	---	Recife	---	10_1929	AC, AM, AP, BA, ES, MG, MS, MT, PA, PE, PR, RJ, RO, SP	s/n	Preserved at Fiocruz-CMN
	---	---		---	1/29/1946		CMN 41379	Preserved at Fiocruz-CMN
Coquillettidia (*Rhynchotaenia*) *chrysonotum* (Peryassú, 1922)	---	---	Recife	---	10_1929	AP, BA, ES, MG, MS, MT, PE, PR, RJ, SC, SE, SP	s/n	Preserved at Fiocruz-CMN
Coquillettidia (*Rhynchotaenia*) *fasciolata* (Lynch Arribálzaga, 1891)	---	---	Recife	---	7/20/1948	AC, AM, AP, BA, ES, GO, MG, MS, MT, PA, PB, PE, PR, RJ, RO, SE, SP, TO	CMN 42799	Preserved at Fiocruz-CMN
	---	---	Ipojuca	---	8/28/1954		CMN 43566	Preserved at Fiocruz-CMN
Coquillettidia (*Rhynchotaenia*) *hermanoi* (Lane & Coutinho, 1940)	---	---	Recife	---	07/2009-11/2009	BA, MG, MS, MT, PA, PE, PR, SC, SP	---	3
Coquillettidia (*Rhynchotaenia*) *juxtamansonia* (Chagas, 1907)	---	---	Recife	---	10_1929	AC, AM, BA, GO, MG, MS, MT, PA, PB, PE, PR, RJ, RN, SC, SE, SP, TO	s/n	Preserved at Fiocruz-CMN
	---	---	Recife	---	4/6/1946		s/n	Preserved at Fiocruz-CMN
	-8.140	-35.084	Moreno	---	09/2007-10/2007		---	5
	-8.124	-35.098	Moreno	---	02/2008-03/2008		---	5

Continue...

Supplementary Table S1: Continuation.

	Latitude	Long	Municipality	Larval Habitats	Registration date	Brazilian states	Voucher	References list
Coquillettidia (Rhynchotaenia) nigricans (Coquillett, 1904)	-8.140	-35.084	Moreno	---	05/2007-06/2007	AM, MG, MS, MT, PA, PE, PR, RJ, RN, RO, RS, SP	---	5
Coquillettidia (Rhynchotaenia) shannoni (Lane & Antunes, 1937)	---	---	Recife	---	07/2009-11/2009	BA, MG, MS, MT, PE, PR, RJ, RS, SC, SE, SP, TO	---	3
Coquillettidia (Rhynchotaenia) venezuelensis (Theobald, 1912)	-8.126	-35.094	Moreno	---	05/2007-06/2007	AC, AM, AP, BA, DF, GO, MG, MS, MT, PA, PE, PR, RJ, RN, RO, RS, SC, SP	---	5
Mansonia (Mansonia) humeralis Dyar & Knab, 1906	-8.042	-35.196	São Lourenço da Mata	permanent water reservoir	6/14/2022	AM, BA, CE, MA, MG, MS, MT, PA, PB, PE, PR, RJ, RN, RO, RS, SP	DZUP 604119	present study
Mansonia (Mansonia) indubitans Dyar & Shannon, 1925	---	---	Petrolina	---	3/4/1933	AC, AM, AP, BA, CE, ES, GO, MA, MG, MS, MT, PA, PE, PR, RJ, RN, RO, SC, SE, SP, TO	CMN 551	Preserved at Fiocruz-CMN
	---	---	Bezerros	---	1/4/1938		CMN 16930	Preserved at Fiocruz-CMN
	-8.140	-35.084	Moreno	---	05/2007-06/2007		---	5
Mansonia (Mansonia) wilsoni (Barreto & Coutinho, 1944)	---	---	Recife	---	07/2009-11/2009	BA, GO, ES, MG, MS, MT, PE, PR, RJ, RN, RO, RS, SC, SP	---	3
Mansonia (Mansonia) titillans (Walker, 1848)	---	---	Recife	---	09_1929	AC, AM, AP, BA, CE, ES, MG, MS, MT, PA, PB, PE, PI, PR, RJ, RN, RO, RS, SC, SE, SP, TO	s/n	Preserved at Fiocruz-CMN
	---	---	Petrolina	---	03_1933		CMN 552	Preserved at Fiocruz-CMN
	-8.140	-35.084	Moreno	---	09/2007-10/2007		---	5
Sabethini				---				
Isostomyia lunata (Theobald, 1901)	---	---	Recife	---	before 1900	BA, GO, PE, RJ, SC	---	1 (as. *Joblotia lunata*)
Limatus durhamii Theobald, 1901	---	---	Recife	---	7/23/1948	AC, AM, AP, BA, DF, ES, GO, MA, MG, MS, MT, PA, PE, PR, RJ, RO, RS, SC, SE, SP, TO	CMN 42811	Preserved at Fiocruz-CMN
	---	---		---	07/2009-11/2009		---	3
Onirion brucei (del Ponte & Cerqueira, 1938)	---	---	Recife	---	1/29/1946	AM, BA, MT, PE, PR, RJ, SP	CMN 41379	Preserved as Wyeomyia brucei at Fiocruz-CMN

Continue...

Supplementary Table S1: Continuation.

	Latitude	Long	Municipality	Larval Habitats	Registration date	Brazilian states	Voucher	References list
Sabethes (*Sabethes*) *albiprivus* Theobald, 1903	---	---	Recife	---	4/1/1946	AM, AP, BA, CE, DF, MA, MG, MS, MT, PA, PE, RJ, RS, SE, SC, SP	CMN 41381	Preserved at Fiocruz-CMN
	---	---		---	7/6/1946		CMN 41902	Preserved at Fiocruz-CMN
Sabethes (*Sabethes*) *purpureus* (Theobald, 1907)	-8.0778	-34.9666	Recife	---	8/8/2022	AM, GO, MG, MS, MT, PE, PR, RJ, RS, SC, SP, TO	DZUP 604135	present study
Sabethes (*Sabethes*) *tarsopus* Dyar & Knab, 1908	---	---	Recife	---	07/2009-11/2009	AM, BA, MS, MT, PA, PE, RJ	---	3
Shannoniana fluviatilis (Theobald, 1903)	---	---	Recife	---	before 1900	BA, ES, GO, PA, PE, PR, RJ, RO, SC, SP	---	1 (as. *Goeldia fluviatilis*)
Trichoprosopon digitatum (Rondani, 1848)	---	---	Recife	---	before 1900	AC, AM, AP, BA, ES, GO, MS, MT, PA, PE, PI, PR, RJ, SC, SP	---	1 (as. *Trichoprosopon nivipes*)
Trichoprosopon lampropus (Howard, Dyar & Knab, 1913)	---	---	Recife	---	07/2009-11/2009	PE	---	3
Wyeomyia negrensis Gordon & Evans, 1922	---	---	Recife	---	07/2009-11/2009	AM, AP, MA, PA, PE, PR, RJ, RR	---	3
Wyeomyia serratoria (Dyar & Nunez Tovar, 1927)	---	---	Recife	---	07/2009-11/2009	MS, PE, PR, RJ, SP	---	3
Wyeomyia (*Dendromyia*) *ypsipola* Dyar, 1922	---	---	Recife	---	1/29/1946	AC, AM, BA, MA, MT, PA, PE, RJ, RO	CMN 41379	Preserved at Fiocruz-CMN
	-8.054	-34.881		---	8/8/2006		CCULI 2901	Preserved at Fiocruz
Wyeomyia (*Phoniomyia*) *incaudata* Root, 1928	-8.015	-34.951	Recife	phytotelm	7/28/2021	BA, GO, MG, PE, PR, RJ, SC, SP	---	present study
	-8.015	-34.948	Recife	bromeliad	9/23/2021		---	present study
	-8.010	-34.948	Recife	bromeliad	9/27/2021		DZUP 604122 to 604124	present study
	-8.043	-35.207	Vitória de Santo Antão	bromeliad	8/15/2021		DZUP 604121	present study
Wyeomyia (*Phoniomyia*) *longirostris* Theobald, 1901	---	---	Recife	---	before 1900	PE, PR, RJ, SC, SP	---	1 (as. *Phoniomyia longirostris*)
Wyeomyia (*Phoniomyia*) *pilicauda* Root, 1928	-8.042	-35.207	Vitória de Santo Antão	bromeliad	6/16/2022	GO, PA, PE, PR, RJ, SC, SP	DZUP 604125	present study
	-8.042	-35.208			6/16/2022		DZUP 604126	present study
Wyeomyia (*Phoniomyia*) *trinidadensis* Theobald, 1901	---	---	Recife	---	1/29/1946	BA, PE, RJ, SE, SP	CMN 41379	Preserved at Fiocruz-CMN
Wyeomyia (*Phoniomyia*) *tripartita* (Bonne-Wepster & Bonne, 1921)	---	---	Recife	---	3/30/1946	PE, GO, RS, SC, SE	CMN 41380	Preserved at Fiocruz-CMN
Wyeomyia (*Spilonympha*) *airosai* Lane & Cerqueira, 1942	-8.043	-35.207	Vitória de Santo Antão	bromeliad	8/15/2021	ES, PE, RJ, SP	DZUP 604127	present study

Continue...

Supplementary Table S1: Continuation.

	Latitude	Long	Municipality	Larval Habitats	Registration date	Brazilian states	Voucher	References list
Wyeomyia (*Spilonympha*) *bourrouli* (Lutz, 1905)	-8.054	-34.881	Recife	---	8/8/2006	AP, BA, ES, MA, MG, PA, PE, RJ, RN, SC, SP	CCULI 2882	Preserved at Fiocruz
Wyeomyia (*Spilonympha*) *mystes* Dyar, 1924	---	---	Recife	---	3/30/1946	AP, ES, GO, MG, MS, MT, PA, PE, PR, RJ, SP	CMN 41380	Preserved at Fiocruz-CMN
Wyeomyia (*Triamyia*) *aporonoma* Dyar & Knab, 1906	---	---	Recife	---	1/29/1946	AM, AP, BA, ES, GO, MG, MS, MT, PA, PE, PR, RJ, RR, RS, SC, SE, SP	CMN 41379	Preserved at Fiocruz-CMN
Wyeomyia (*Wyeomyia*) *arthrostigma* (Lutz, 1905)	-8.054	-34.881	Recife	---	8/8/2006	BA, ES, MA, MT, PA, PE, PR, RJ, RO, SE, SP	CCULI 2900	Preserved at Fiocruz
Wyeomyia (*Wyeomyia*) *medioalbipes* Lutz, 1904	---	---	Recife	---	1/29/1946	AL, AM, AP, BA, CE, ES, GO, MG, MS, MT, PA, PE, RJ, RR, SC, SP	CMN 41379	Preserved at Fiocruz-CMN
	-8.054	-34.881		---	8/8/2006		CCULI 2895	Preserved at Fiocruz
Uranotaeniini								
Uranotaenia (*Uranotaenia*) *apicalis* Theobald, 1903	-8.013	-35.204	São Lourenço da Mata	permanent water reservoir	8/16/2021	AC, AM, MT, PE, PR, SE, SP, TO	DZUP 604128	present study
	-8.026	-35.204		fish tank	6/28/2022		DZUP 604129	present study
Uranotaenia (*Uranotaenia*) *geometrica* Theobald, 1901	---	---	Recife	---	10_1929	AM, BA, CE, ES, GO, MG, MS, MT, PA, PE, PR, RJ, RO, RS, SP	s/n	Preserved at Fiocruz-CMN
Uranotaenia (*Uranotaenia*) *lowii* Theobald, 1901	---	---	Recife	---	10_1929	AM, BA, ES, MG, MS, MT, PE, PR, RJ, RN, RS, SC, SE, SP	s/n	Preserved at Fiocruz-CMN
	---	---		---	06_1930		CMN 671	Preserved at Fiocruz-CMN
Uranotaenia (*Uranotaenia*) *pulcherrima* Lynch Arribálzaga, 1891	---	---	Recife	---	09_1929	AM, BA, ES, GO, MG, MS, MT, PE, PR, RJ, RR, RS, SC, SP, TO	s/n	Preserved at Fiocruz-CMN
Toxorhynchitini								
Toxorhynchites (*Lynchiella*) *theobaldi* (Dyar & Knab, 1906)	-7.924	-35.165	Paudalho	---	5/1/2021	CE, DF, MG, PE, PR, RS, SC, SP	76733077	Human observation at iNaturalist

References list. 1: Bourroul (1904); **2:** Albuquerque et al., 2000; **3:** Aragão et al., 2010; **4:** Ramos et al., 2011; **5:** da Silva et al., 2018; **6:** Deane et al., 1948. **CEIOC:** Entomological Collection of the Oswaldo Cruz Institute; **CMN:** Neotropical Mosquito Collection; **DZUP:** Padre Jesus Santiago Moure Entomological Collection. **FMNH:** Field Museum of Natural History; **USNM:** Smithsonian National Museum of Natural History. **AC:** Acre; **AL:** Alagoas; **AM:** Amazonas, **BA:** Bahia; **CE:** Ceará; **DF:** Distrito Federal; **ES:** Espírito Santo; **GO:** Goiás; **MG:** Minas Gerais; **MT:** Mato Grosso; **MS:** Mato Grosso do Sul; **PA:** Pará; **PE:** Pernambuco; **PR:** Paraná; **RJ:** Rio de Janeiro; **RN:** Rio Grande do Norte; **RO:** Rondônia; **RR:** Roraima; **RS:** Rio Grande do Sul; **SC:** Santa Catarina; **SE:** Sergipe; **SP:** São Paulo; **TO:** Tocantins.

REFERENCES:

1. Bourroul C. Mosquitos do Brasil. Salvador: Faculdade de Medicina da Bahia; 1904.
2. Albuquerque CM de, Melo-Santos MAV, Bezerra MAS, Barbosa RM, Silva DF, Silva E da. Primeiro registro de *Aedes albopictus* em área da Mata Atlântica, Recife, PE, Brasil. Rev Saude Publica 2000;34(5):3–5.
3. Aragão NC, Müller GA, Balbino VQ, Costa Junior CRL, Figueirêdo Júnior CS, Alencar J, et al. A list of mosquito species of the Brazilian State of Pernambuco, including the first report of *Haemagogus janthinomys* (Diptera: Culicidae), yellow fever vector and 14 other species (Diptera: Culicidae). Rev Soc Bras Med Trop 2010;43(4):458–9.
4. Ramos RAN, Costa GJA, Carvalho GA de, Faustino MAG, Alves LC. First Record of *Culex* (*Culex*) *maxi* Dyar (Diptera: Culicidae) in Pernambuco State, Brazil. Arq Inst Biol (São Paulo) 2011;78(4):623–4.
5. da Silva CJ, Pereira SV, Apolinário EJ, dos Santos GL, Melo-Santos MAV, da Silva AF, et al. Culicidae fauna (Diptera: Culicidae) survey in urban, ecotonal and forested areas, from the Moreno municipality - Pernambuco State, Brazil. Rev Soc Bras Med Trop 2018;51(4):523–7.
6. Deane LM, Ausey OR, Deane MP. Notas sobre a distribuição e a biologia dos anofelinos das regiões nordestina e amazônica do Brasil. Rev Do Serviço Espec Saúde Pública 1948;1(4):827–965.
